# Past Fire and Vegetation Change in the Hyperdiverse Forests of the Ecuadorian Amazon

**DOI:** 10.3390/plants13152048

**Published:** 2024-07-25

**Authors:** Britte M. Heijink, Annabel Zwarts, Nina H. Witteveen, Jessica Watson, Arie Ebbenhorst, Fedde Veenman, Mats Kessel, Susana León-Yánez, Juan Ernesto Guevara-Andino, María-José Endara, Gonzalo Rivas-Torres, Mark B. Bush, Crystal N. H. McMichael

**Affiliations:** 1 Department of Ecosystem and Landscape Dynamics, Institute for Biodiversity and Ecosystem Dynamics, University of Amsterdam, 1012 WP Amsterdam, The Netherlands; britteheijink@outlook.com (B.M.H.); azwarts2023@my.fit.edu (A.Z.); n.h.witteveen@uva.nl (N.H.W.); arie.ebbenhorst@gmail.com (A.E.); feddeveenman@kpnmail.nl (F.V.); kesselmats@gmail.com (M.K.); 2 Institute for Global Change, Florida Institute of Technology, Melbourne, FL 32901, USA; watson2021@my.fit.edu (J.W.); mbush@fit.edu (M.B.B.); 3 Escuela de Ciencias Biológicas, Pontificia Universidad Católica del Ecuador, Quito 170143, Ecuador; scleon@puce.edu.ec; 4 Grupo de Investigación en Ecología y Evolución en los Trópicos-EETrop, Universidad de las Américas, Quito 170124, Ecuador; jguevara@fieldmuseum.org (J.E.G.-A.); maria.endara.burbano@udla.edu.ec (M.-J.E.); 5 Estación de Biodiversidad Tiputini, Colegio de Ciencias Biológicas y Ambientales, Universidad San Francisco de Quito (USFQ), Quito 170901, Ecuador; grivast@usfq.edu.ec

**Keywords:** charcoal, fire, forest management, human influence, hyperdominant, paleoecology, palms, phytoliths, tropical forests

## Abstract

The Ecuadorian Amazon holds more biodiversity than most other places on Earth. Palms are a particularly dominant component of the vegetation; however, it remains unknown to what degree the pattern has persisted through time. Here, we investigate the persistence of palm dominance through time and the degree to which past human activities (e.g., fire, cultivation, and forest opening) have affected changes in palm abundances across five regions of the Ecuadorian Amazon. We analyzed soil cores (40–80 cm depth) from each region for charcoal (evidence of past fire) and phytoliths (evidence of past vegetation change). The timings of fires (based on ^14^C radiocarbon dates), the occurrence, recurrence, and number of fires (based on charcoal presence and abundance in samples), and the amount of change in palm abundances (based on phytoliths) varied within and between the studied regions. The charcoal and phytolith results indicate the presence of low levels of past human activity at all sites. Our results show that patterns of modern palm hyperdominance found in Amazonian forests have not been persistent through time, and that even low levels of past human activities can affect palm abundance.

## 1. Introduction

Plant species richness in Amazonia generally follows precipitation gradients, where the wettest forests are more speciose than the drier ones [1,2,3]. Species richness is highest in northwestern Amazonia, which has the highest annual precipitation (up to 4000 mm) and fewest number of dry months (receiving less than 100 mm/month) compared with other regions of the basin [3]. This pattern is also consistent over evolutionary time with current evidence suggesting that peaks of phylogenetic diversity (PD) follow a west–east gradient in precipitation and are particularly high in the northwestern Amazon [4]. The Yasuní region of northwestern Amazonia is one of the most diverse areas on Earth, containing over 5487 tree species, over 600 bird species, and over 200 mammal species per square kilometer [5]. In addition, both PD and Evolutionary Distinctiveness (AED) are remarkably high in this region compared to other regions in the Ecuadorian Amazon [6]. 

This extraordinary diversity is paired with patterns of tree dominance at both continental and regional scales [7,8]. Of the estimated 16,000 tree species present in Amazonia, ca. 227 hyperdominant species (1.5% of the total estimated species) account for half of the estimated individual tree stems [8,9,10]. These hyperdominant species play a large role in shaping ecosystem functioning, e.g., fruit provisioning [11] and services, e.g., carbon sequestration [12,13]. Of the 227 hyperdominant species, 15 are palms (the Arecaceae family), which is five-times more than expected by chance [8]. The disproportionate importance of palms is a characteristic of Neotropical forests as their relative abundance is significantly higher compared with African or Asian rainforests [14]. Both abiotic factors, such as microtopography and soil types, and biotic factors, such as seed predation and dispersal, play a role in shaping the observed patterns of palm species richness and relative abundance [15,16,17,18,19,20,21,22,23]. Palms are widely used in tropical forests, and humans influence their abundance and distribution [24,25,26,27,28,29].

The persistence of Amazonian palm hyperdominance through time remains unknown. Palms have been present in Amazonian landscapes for millions of years [30,31], but their relative abundances have likely changed over time [27,32]. Pollen-based paleoecological reconstructions have shown that *Iriartea deltoidea*, a palm that is currently the fifth most common tree in the Amazon [8], has increased in abundance in forests around 13 western Amazonian lakes over the last 3000 years [32]. Lake sediment records containing high levels of past fire and human activity had continuously low levels of *Iriartea* pollen, and increases in abundances occurred mainly at sites containing low levels of human activity, i.e., infrequent fires, low-level forest opening, and infrequent maize cultivation [32,33,34]. *I. deltoidea*’s increases in abundance are not coincident with human activity, but often occur several hundred years following forest burning by pre-Columbian Indigenous people [34]. This is attributed to increased amounts of precipitation combined with reduced levels of from human exploitation [34].

The areas around lakes are preferred settlement sites for people both in the past and in the present, and almost all lake sediment records analyzed show evidence of past human activity at some point during the Holocene [35,36,37]. Lake sediment records are thus particularly useful for assessing the impact of past human activity on palm abundance. Amazonia, however, contains relatively few permanent lakes that can provide these reconstructions [38]. Soils can be sampled from anywhere in terrestrial environments and can be used to compare the histories of fire and vegetation changes in sites ranging from preferred to marginal. Pollen, however, degrades in soils. Phytoliths, which are silica microfossils produced by many Neotropical plant families [39], can be analyzed from terrestrial soil cores to provide a localized signal (around 10 s to 100 s of meters) of vegetation change [40,41]. Phytoliths and charcoal derived from terrestrial soil cores have been used to indicate local-scale fire events and vegetation change in Amazonian forests over the last 5000 years [39,42]. Phytoliths can be used to detect the cultivation of plants such as maize (*Zea mays*), gourds, and squashes (Cucurbitaceae), lerén (*Calathea allouia*), arrowroot (*Maranta* spp.), rice (*Oryza* spp.), and bananas (*Musa* spp., which were cultivated following the arrival of Europeans) [39,43,44,45]. Phytoliths are also particularly sensitive to detecting various types of monocotyledon plants, such as grasses and other early successional plants (e.g., Zingiberales, Asteraceae), which indicate forest openings [39,46]. Palms are monocotyledonous plants and prolific phytolith producers, and the various palm phytolith morphotypes can often be assigned to specific palm genera [47,48].

**Figure 1:**
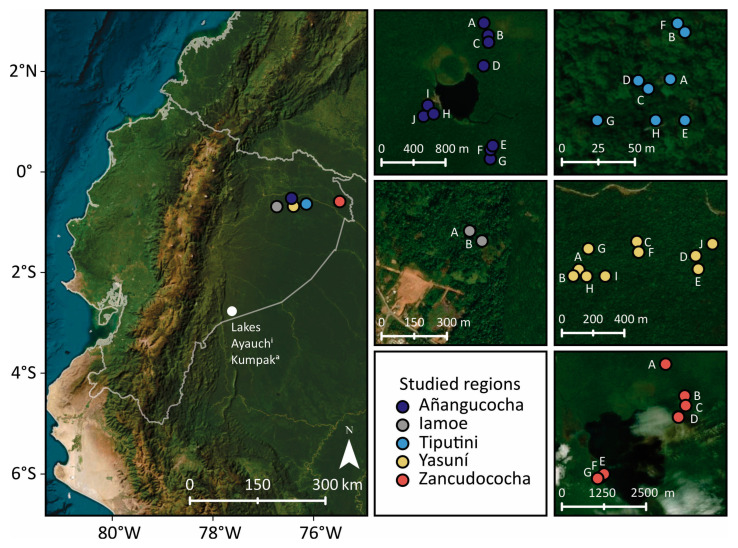
Map of the studied regions. The left panel shows the location of the studied regions in Ecuador and other sites mentioned in the text. The smaller panels show the locations of soil cores (labeled with letters that correspond with Figures 3–7) collected in each study region.

Northwestern Amazonia contains the highest palm diversity (and potentially abundances) in the Basin. There are an estimated 64 species of palms in northwestern Amazonia that can comprise 30–40% of the total individuals measured in vegetation surveys [20]. There is also the long-term human occupation of the region, and maize has been cultivated in some areas for at least 6000 years [49]. Many areas of northwestern Amazonia, however, contain little to no past human footprints [42]. The influence of past human activity on palm abundance is thus unclear. Phytoliths and charcoal collected in the Amacayacu forest plot of Colombia and in the Yasuní forest plot of Ecuador (both in northwestern Amazonian forests), where modern vegetation was also studied, showed localized increases in *Iriartea* (a common canopy palm) abundances that did not appear to correspond with past fire events or past human activities [50,51]. Here we expand on the phytolith and charcoal analyses from terrestrial soil cores across the hyperdiverse Ecuadorian Amazon [50] by generating data from four new localities (Figure 1). We use the charcoal and phytolith data to assess the variability in local-scale change in fire and palm abundances across the studied sites in the Ecuadorian Amazon. We specifically ask whether: (i) the timing of past fire events is synchronous across sites, (ii) the variance in phytolith assemblages is uniform or heterogeneous across the Ecuadorian Amazon, or (iii) the changes in the abundances of palms or arboreal taxa through time are associated with past cultivation or fire occurrence. 

## 2. Results

### 2.1. Fire History of the Ecuadorian Amazon

**Figure 2:**
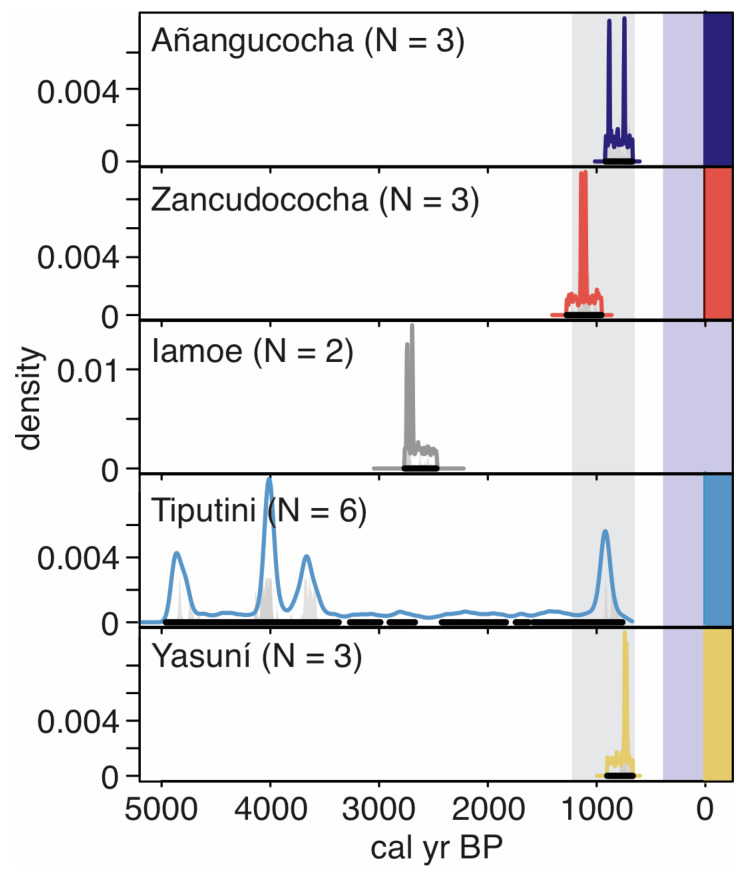
Density diagram of dated charcoal fragments (colored lines for each site showing the most likely ages of fires) from the five surveyed regions in the Ecuadorian Amazon. Site colors are based on the colors used in the map of the study region (Figure 1). Black lines underneath each set of ages represent the range of ages for each date. The light-gray vertical bar indicates the period of synchronous fire events in the pre-Columbian period, and the light-purple vertical bar indicates the times following Europeans’ arrival in the northwestern Amazonian rainforests. Vertical bars at 0 cal BP (calibrated years before present) indicate modern ^14^C dates that could result from fires occurring anywhere from AD 1950 until the year of collection (Table 1). See Table 1 for additional details on ^14^C ages from each site.

Evidence of past fires was found in all the sampled regions, and charcoal fragments large enough for ^14^C AMS dating were found in each region (Table 1 and Figure 2). Four of five regions contained evidence of modern fire, and those same four regions showed synchronicity in the timings of pre-Columbian fire events in ca. 800–1200 calibrated years before present (hereafter cal BP) (Figure 2). Iamoe was the only site that did not contain evidence of modern fire or fires in the 800–1200 cal BP range, and the time since the last recorded fire was ca. 2670 cal BP (Table 2). Most of the obtained ^14^C ages were from the last 3000 years, though Tiputini contained evidence of fire events as early as 4800 cal BP.

**Figure 3:**
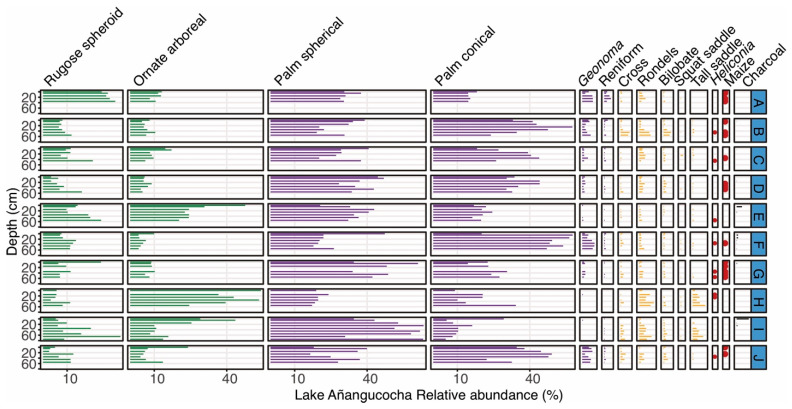
Phytolith types shown by depth intervals for the 10 cores (A–J) collected around Lake Añangucocha. Green phytolith types represent arboreal taxa, purple types represent palm taxa, and yellow types represent various types of grasses. The presence of *Heliconia* and maize (*Zea mays*) phytoliths are shown as red dots. Charcoal abundances (mm^3^/cm^3^) are shown in black.

**Figure 4:**
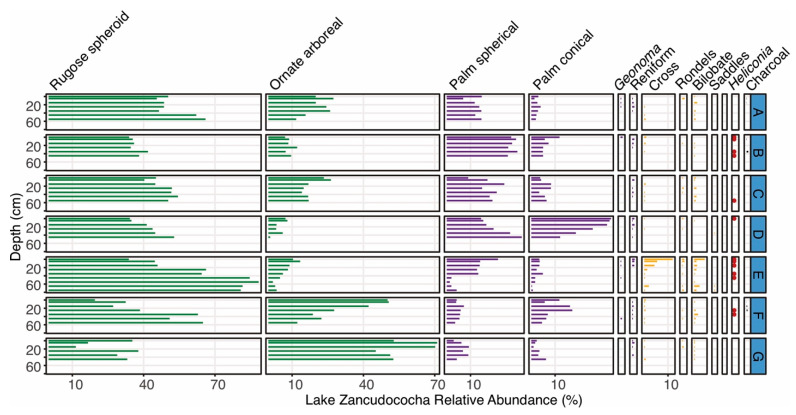
Phytolith types shown by depth intervals for the 7 cores (A–G) collected around Lake Zancudococha. Green phytolith types represent arboreal taxa, purple types represent palm taxa, and yellow types represent various types of grasses. The presence of *Heliconia* phytoliths is shown as red dots. Charcoal abundances (mm^3^/cm^3^) are shown in black.

**Figure 5:**
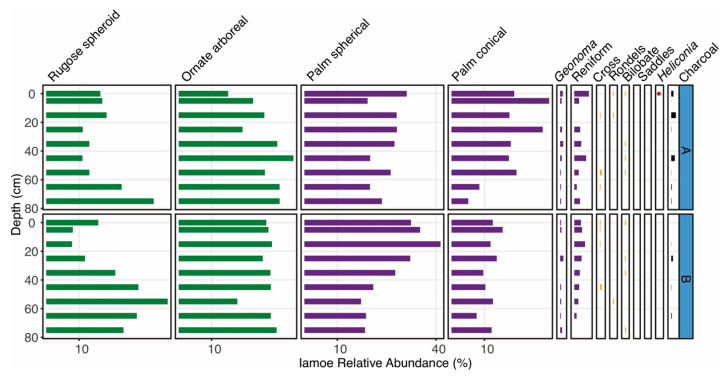
Phytolith types shown by depth intervals for the 2 cores (A,B) collected around the Iamoe forest plot. Green phytolith types represent arboreal taxa, purple types represent palm taxa, and yellow types represent various types of grasses. The presence of *Heliconia* is shown as red dots. Charcoal abundances (mm^3^/cm^3^) are shown in black.

**Figure 6:**
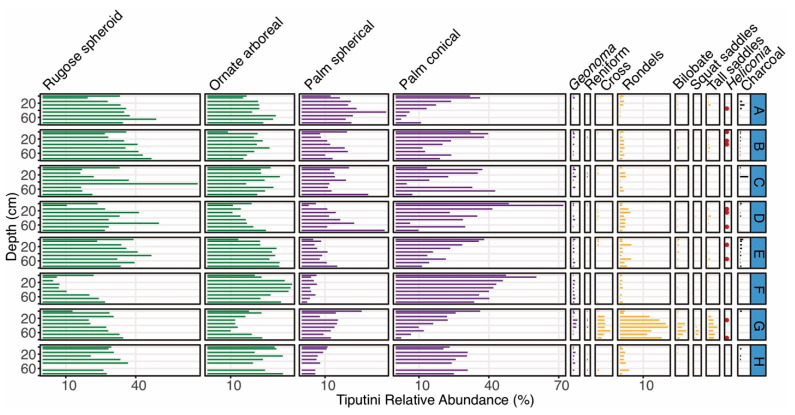
Phytolith types shown by depth intervals for the 8 cores (A–H) collected around the Tiputini forest plot. Green phytolith types represent arboreal taxa, purple types represent palm taxa, and yellow types represent various types of grasses. The presence of *Heliconia* is shown as red dots. Charcoal abundances (mm^3^/cm^3^) are shown in black.

**Figure 7:**
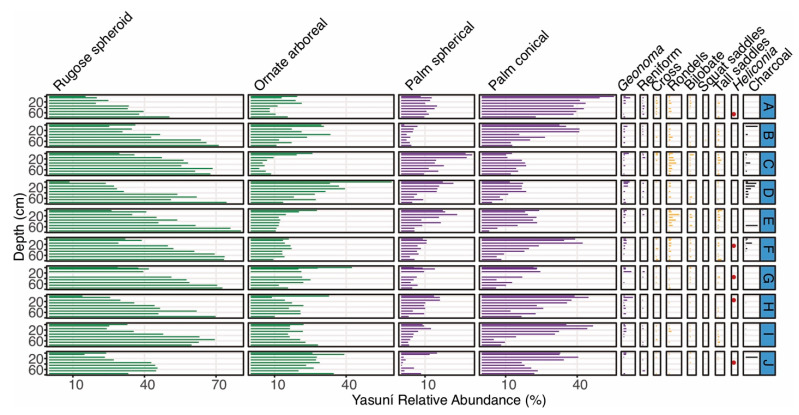
Phytolith types shown by depth intervals for the 10 cores (A–J) collected around the Yasuní forest plot. Green phytolith types represent arboreal taxa, purple types represent palm taxa, and yellow types represent various types of grasses. The presence of *Heliconia* is shown as red dots. Charcoal abundances (mm^3^/cm^3^) are shown in black.

The two sites with less than 2 hectares surveyed had higher proportions of cores and samples containing charcoal (Table 2). With the exception of the Lake Añangucocha area, all of the regions contained charcoal in over half of the soil cores analyzed (Table 2). None of the 58 soil cores analyzed contained uniformly high abundances of charcoal or recurrent layers of large and datable charcoal fragments throughout the soil cores (Table 2 and Figure 3, Figure 4, Figure 5, Figure 6 and Figure 7).

### 2.2. Local-Scale Patterns of Vegetation Change in the Ecuadorian Amazon

Evidence of past cultivation was only found at Lake Añangucocha (Figure 3). Seven of the 10 cores collected around Lake Añangucocha contained maize phytoliths (21 total samples) (Figure 3), and those seven cores were located on the north and south sides of the lake (Figure 1). Charcoal was present in five of the eight cores containing maize phytoliths, and Poaceae percentages were in the range of 1–19% in those eight cores. The three cores that did not contain evidence of maize cultivation lacked charcoal, and Poaceae percentages in those cores were in the range of 1–13% (Figure 3). The cores that lacked evidence of maize cultivation contained the highest percentages of arboreal phytoliths (both the rugose spheroid and ornate forms), whereas the cores containing maize phytoliths had lower arboreal percentages but higher percentages of conical palm phytoliths, which are produced by genera such as *Iriartea* and *Socratea*. 

Though maize phytoliths were not found at Zancudococha, charcoal was present in two of the seven soil cores (Figure 4). *Heliconia* occurred in five of the seven cores, but was most frequent in Core Zan-E, which also contained high (20%) percentages of Poaceae phytoliths in the surface sample (Figure 4). Core Zan-E was located on the northeast side of the lake, where lodge construction was occurring (Figure 1). The percentages of arboreal phytoliths were typically higher and the percentages of palm phytoliths were generally lower around Lake Zancudococha compared with Lake Añangucocha (Figure 3 and Figure 4). In cores Zan-E and Zan-F (Figure 1 and Figure 4), the abundances of rugose spheroid arboreal phytoliths decreased over 40%. This decrease was accompanied by increases in the abundances of other arboreal phytolith morphotypes in one core, and by increases in the abundances of spherical echinate palm phytoliths, which are produced by *Oenocarpus* and *Attalea* palm genera, in the other core (Figure 4).

The two cores in the two hectares of the Iamoe forest plot had low charcoal abundances and lacked cultivars. Poaceae phytoliths were less than 5% of the overall assemblage in all samples, though *Heliconia* was present in the surface sample of Core Iam-A. Overall palm abundances were similar to those found at Lake Añangucocha, though Poaceae abundances were lower (Figure 3 and Figure 5). Both Iamoe cores contained decreases of over 20% rugose spheroid arboreal phytoliths through time. Core Iam-A showed a >20% increase in conical palm phytoliths, and Core Iam-B contained a > 20% increase in spherical phytoliths. Vegetation surveys at Iamoe indicated that *Iriartea deltoidea* is the most common palm species that produces conical palm phytoliths, and *Oenocarpus bataua* is the most common species that produces spherical palm phytoliths.

Seven of the eight cores collected in the Tiputini forest plot contained charcoal (Figure 6), including six fragments that were large enough for ^14^C dating (Table 1 and Table 2). No cultivar phytoliths were found. Poaceae phytoliths were generally less than 5% of the overall assemblage in all cores except Core Tip-G, where all samples below a 10 cm depth had abundances exceeding 20% (Figure 6). *Heliconia* was present in Core Tip-G and in four other cores that contained lower Poaceae abundances (Figure 6). Most of the Tiputini soil cores contained increases in conical palm phytoliths, but there were no clear trends of spherical palm phytoliths. Vegetation surveys at Tiputini show that *Iriartea deltoidea* (which produces conical palm phytoliths) and *Oenocarpus bataua* (which produces spherical palm phytoliths) are the two commonest palms in the area [6,7,53,54].

Six of the ten cores analyzed in the Yasuní forest plot contained charcoal, including three fragments that were large enough for ^14^C dating (Table 1 and Table 2) [50]. No cultivar phytoliths were found. Poaceae phytoliths were generally less than 10% of the overall assemblage in all cores (Figure 7). *Heliconia* was present in five cores, including three of the four that lacked charcoal (Figure 7). Seven of the ten Yasuní soil cores contained increases in conical palm phytoliths, and six out of ten contain increases in spherical phytolith types through time [50]. Vegetation surveys at Yasuní show that *Iriartea deltoidea* is the most common palm species (which produces conical palm phytoliths), followed by *Oenocarpus bataua* and *Euterpe precatoria* (which produce spherical palm phytoliths) [6,7,53,55,56].

### 2.3. Regional Trends and Patterns of Vegetation Change in the Ecuadorian Amazon

**Figure 8:**
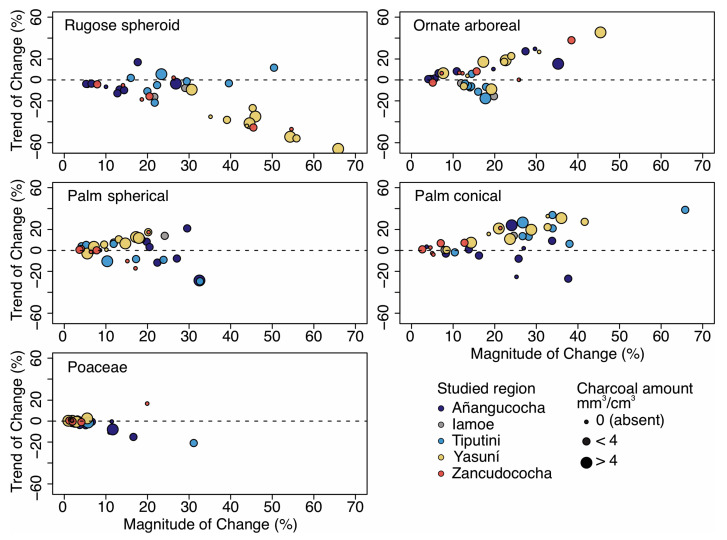
Magnitude of change versus trend of change metrics for various phytolith types across the six studied regions. Each point represents the difference in phytolith percentage between the uppermost and lowermost depth intervals (y-axis), and the difference between the maximum and minimum phytolith percentage within a core (x-axis). Each core is color-coded by study region, and the size of the point represents the total amount of charcoal found in each core.

The trends and magnitudes of change varied among arboreal phytolith types across the five study regions. Rugose sphere arboreal phytoliths decreased in abundance from the bottom to the top of the core, i.e., the trend of change was negative, and the decrease was largest (i.e., high magnitude of change values) in the Yasuní forest plot and in a couple of cores collected around Lake Zancudococha (Figure 8). The Tiputini forest plot also had high magnitude of change values (i.e., highly varying percentages) for rugose spheroid phytoliths, though there was no trend through the depths of the cores (Figure 8). Several cores at Lake Añangucocha and in the Yasuní forest plot showed large-scale increases (15–45%) of ornate spheroid phytoliths, and several cores at the Tiputini forest plot showed low magnitude (10–20%) decreases.

The trends and magnitudes of change in palm phytoliths were larger in the conical phytoliths than in the spherical palm phytoliths (Figure 8). Conical palm abundances exhibited large changes (>20% magnitude of change) in the positive direction in at least one core from every site, but only at Lake Añangucocha in the negative direction (Figure 8). There were no clear inter-regional patterns of magnitude of change or trend of change values for the spheroid palm phytoliths. Though the magnitude of change values exceeded 15% in at least one core from each region, the direction was not consistent within a region (Figure 8).

Most regions experienced very little change in Poaceae phytoliths, either in magnitude or direction (Figure 8). Several cores around Lake Añangucocha and one core at the Tiputini forest plot had high magnitude of change values (i.e., high variability in percentages) with negative trend of change values, indicating forests closing (reforesting) through time. One core around Lake Zancudococha (Zan-E) showed a high magnitude of change (18%) with positive trend of change values (i.e., forest opening through time).

**Figure 9:**
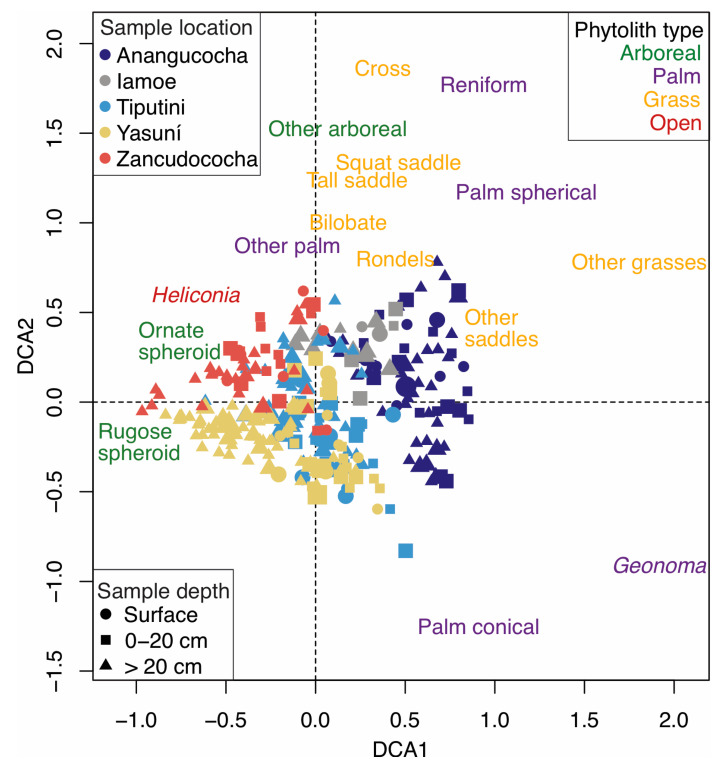
Detrended correspondence analysis of phytolith assemblages from the studied regions. Points indicate the positions of the individual phytolith samples on the ordination, and the taxa scores of the phytolith morphotypes are shown in black text. Samples are color-coded by region, and symbols indicate the depths of the samples. The size of the symbol represents the amount of charcoal found in the sample.

Phytolith assemblages were not unique amongst the five studied regions. The detrended correspondence analysis (DCA) showed that there was an overlap in the phytolith composition between most regions, except Lake Añangucocha (Figure 9). The phytolith assemblages from the forests around Lake Añangucocha were located on the far positive side of DCA Axis 1 and contained higher abundances of grasses (i.e., saddle and rondel morphotypes) and palm phytoliths compared with other regions (Figure 3, Figure 4, Figure 5, Figure 6, Figure 7 and Figure 9). DCA Axis 2 separates sites based on the abundances of different types of palm phytoliths. Sites with higher abundances of conical palm phytoliths are located on the positive end of Axis 2, and sites with higher abundances of spheroid palm phytoliths are located on the negative end (Figure 9). 

## 3. Discussion

### 3.1. Were Fire Events Synchronous across Regions?

Age–depth relationships can usually be established in lake sediment records because of stratigraphic integrity, but bioturbation, root penetration, etc., typically prevent age–depth relationships from being established in soil archives [57]. To assess the synchronicity of past fire events, we dated individual charcoal fragments that were large enough for ^14^C AMS dating (Table 1). The Tiputini Biological Research Station had the highest number of datable fragments (n = 6), but covered a relatively small area (1 hectare), indicating that the area had burned multiple times over the last several thousand years. Four of the six dated fragments documented fire events that occurred between ca. 4800 and 3600 calibrated years before present (cal BP) (Table 1). No other region surveyed had evidence of fire events during this period. Iamoe also contained evidence of ancient fires, but the only two datable fragments we recovered probably originated from the same fire event that occurred ca. 2700 cal BP. Though our other study sites (Lakes Añangucocha and Zancudococha and Yasuní) did not contain any evidence of fires this old (Figure 2), other regions in northwestern Amazonia burned occasionally between ca. 3000 and 1000 cal BP. Most charcoal fragments recovered from soil cores around Lake Ayauch^i^ in the forests of southern Ecuador (Figure 1) were dated between 3000 and 1000 cal BP [58]. Most charcoal fragments recovered from the Medio Putumayo-Algodon and Tapiche Blanco regions of northern Peru, and the Amacayacu region of the Colombian Amazon also had ages of ca. 3000–1000 cal BP [51,59,60]. These soil charcoal data and charcoal histories reconstructed from lake sediment cores [35] collectively suggest that people were living in and burning northwestern Amazonian forests in the mid-to-late Holocene, but that the fire activities were extremely localized and not synchronous across the landscape. 

Fire events within our studied sites became more synchronous across the landscape ca. 1200–800 cal BP (Figure 2). This period is believed to be the peak of pre-Columbian population size and forest modification [37,61], and also corresponds with the Medieval Climate Anomaly (a period of dryness in this region) [62]. In AD 1541 (ca. 400 cal BP), Europeans traversed the northwestern Amazonian landscape for the first time under the command of Francisco Orellana [63]. Noticeably, all five of our studied sites lack fires immediately prior to this expedition (Figure 2), and all sites except Lake Zancudococha would have been on its path. These data agree with the written accounts of Friar Gaspar de Carvajal, who chronicled the expedition, and wrote about starving and eating shoe leather during the travels down the River Napo and the upper Amazon [63]. 

Four of our five studied regions contained evidence of modern fire (here, all dated charcoal from fire events before AD 1950 are considered modern) (Figure 2). Although the forests around Tiputini and Yasuní Biological Research Stations hold some of the highest biodiversity on Earth [5,56,64], our results suggest that low levels of infrequent fires and disturbances may play a role in maintaining such high levels of species richness [65]. In both Tiputini and Yasuní, fires were likely very localized as the dates were only recovered from a single location. At Yasuní, the modern charcoal date comes from the southwestern corner of the 50-ha forest plot, which is known to be a secondary forest [50,55]. At Tiputini, the forest does not appear to be recently burned. Thus, the modern charcoal date may be derived from a campfire or other contained fires that happened during the oil exploration period, which were common in the area [66]. Interestingly, no modern fires were documented at the Iamoe forest plot (Figure 2). The plot, however, lies in the Waorani Reserve area and Yasuní National Park Buffer Zone. People have occupied the area for the last 60–80 years at least. It is possible that people were predominantly using fire-free forms of cultivation and land management, or that fires during this period were started in the larger region but not in the locality of the forest plot.

### 3.2. Past Cultivation and Forest Opening in the Ecuadorian Amazon

At the Tiputini and Yasuní forest plots, evidence of past maize cultivation was absent, though evidence of canopy opening (via the presence of *Heliconia* phytoliths alongside increased abundances of Poaceae phytoliths) was present in ca. half of the soil cores analyzed (Figure 6 and Figure 7). The magnitude of canopy openings at these sites was generally small (<10% change), and there were no overall trends of forest openings or closings throughout the cores except for Tiputini Core G, which indicated a much more open landscape in the past that became more forested in modern times (Figure 6 and Figure 8). The extremely high levels of Poaceae and the presence of *Heliconia* in this core do not coincide with the presence of fires and may be the result of the terrestrialization of former riverine systems (e.g., drying of former oxbow lakes). The other signals of forest openings in these two forest plots often occurred alongside the presence of charcoal (fire) (Figure 6 and Figure 8). In the aseasonal forests of the Ecuadorian Amazon, fires rarely spread without human ignition [67]. It is, however, not impossible that some of the openings were due to natural gap dynamics, which have a high frequency in western Amazonia [68,69,70]. Treefall gaps due to blowdowns from storms are particularly frequent in the Yasuní National Park area of northwestern Amazonia [56]. Though it should be noted that these blowdowns are not naturally associated with fire. It is interesting, however, that both the Yasuní and Tiputini forest plots have much higher levels of *Heliconia* and Poaceae phytoliths compared with the Amacayacu forest plot, located ca. 700 km to the southeast (though still located in the aseasonal northwestern Amazonian forests). Only six Poaceae phytoliths were recovered in scans of thousands of phytoliths from Amacayacu, and no sign of any type of forest opening was found, even in the presence of low-intensity fires that occurred in 1600–2600 cal BP [51].

At the two lake sites, the forests were also opened in the past (Figure 3 and Figure 4). At Lake Zancudococha, the soil cores located nearest the lake (Cores B–F) have more evidence of past forest openings (i.e., the presence of *Heliconia* phytoliths and increased abundances of Poaceae phytoliths) compared with those farther away from the lake (Figure 1 and Figure 4). Cores B–G at Zancudococha are all located within 1 km of the lake, whereas Core A is located over 2 km away from the lake and lacks *Heliconia* phytoliths (Figure 1 and Figure 4). Core E at Lake Zancudococha had high percentages (>10%) in the surface sample, and the core was collected near a site near the lake edge (Figure 1) that had recently been cleared for the construction of an ecolodge. No evidence of maize phytoliths were found at Lake Zancudococha. 

The forests around Lake Añangucocha were more disturbed than those around Lake Zancudococha, and evidence of past maize cultivation was found in seven out of 10 cores (Figure 3). Cores that contained evidence of maize almost always had the corresponding presence of *Heliconia* phytoliths. The percentages of Poaceae phytoliths were highest in Cores H–J (Figure 3), which are closest to the lake (Figure 1). The amounts of forest openings and cultivation seen around Lake Añangucocha were similar to those documented in soil cores around Lake Ayauch^i^ in the southeastern Ecuadorian Amazon [58]. The lake sediment record from Lake Ayauch^i^ contained a 6000 year history of maize cultivation, and a recent high-resolution pollen and charcoal analysis from the sediment record has shown that forest burning and maize cultivation were almost continuous over the last 2000 years [33,49]. The lake sediment record around Lake Añangucocha contained a spike of *Cecropia* (an early successional, open-forest clade) and the disappearance of the palms *Iriartea* and *Mauritia* ca. 830 cal BP [71]. The timing of these changes in the lake sediment record was coincident with the timing of forest burning around soil Core G (Table 1, Figure 2), which also contained an abundance of maize phytoliths and open-forest indicators (Figure 3). It is possible that the cultivation and forest openings that occurred in the other sampled locations around Lake Añangucocha (Figure 1 and Figure 3) happened ca. 830 cal BP, but it is not certain because of the lack of datable charcoal fragments at these other locations. The original pollen analysis from the Lake Añangucocha sediment core did not include scans for maize pollen grains or charcoal analyses [71], so the continuity and timing of fires and cultivation cannot be pinpointed yet. Newly collected sediment cores with pollen, phytolith, and charcoal analyses from this lake will shed light on the timing and continuity of forest openings and cultivation, which can then be aligned with the patterns seen in our soil cores. 

### 3.3. The Role of Humans in Shaping Palm Abundances and Vegetation Patterns in the Ecuadorian Amazon

Based on the phytolith analyses from the 37 total soil cores spread across the five studied areas, past vegetation change has occurred heterogeneously (i.e., non-uniformly) on both localized (Figure 3, Figure 4, Figure 5, Figure 6 and Figure 7) and regional (Figure 9) spatial scales. The biggest regional difference in phytolith assemblages is based on the amount of disturbance. Lake Añangucocha experienced the most forest openings and maize cultivation in the past, is located on the positive end of DCA Axis 1, and is the most dissimilar from the other sites (Figure 9). The less-disturbed sites have more overlaps and less dissimilarity between them (Figure 9). 

Within the studied regions, however, there has also been significant amounts of vegetation turnover through time. For example, significant amounts of turnover in tree taxa, as evidenced by the high (>20%) magnitude of change and trend of change values of arboreal phytoliths, occurred in almost all regions (Figure 8). The trend is especially strong in the Yasuní forest plot, where decreasing amounts of rugose sphere phytoliths have been replaced with increasing amounts of ornate arboreal phytoliths (Figure 8). At Tiputini, several cores had large fluctuations in rugose arboreal sphere phytoliths, but no general trend. There was, however, a trend at Tiputini that ornate arboreal spheroid phytoliths decreased from the past to the present. The observed patterns of turnover in arboreal taxa seen at both sites (and at Lake Añanguococha) was often associated with high amounts of charcoal in the cores in which it happened (Figure 8). Many times, arboreal phytolith types cannot be attributed to specific genera or species [39]. Rugose spheroid phytoliths, however, are produced by the genera *Aphelandra* (Acanthaceae), *Apidosperma* (Apocynaceae), some species of the genus *Protium* (Burseraceae), *Acalypha* (Euphorbiaceae), *Vismia* (Hypericacaceae), many species of *Eschweilera* (Lecythidaceae), and several species in the Chrysobalanaceae, Malvaceae, Moraceae, and Rutaceae families [72,73]. Ornate arboreal phytoliths are produced by members of the Acanthaceae, Apocynaceae, Burseraceae, Euphorbiaceae, Lecythidaceae, Malvaceae, Moraceae, Violaceae, and Vochysiaceae families [72,73]. While we cannot say with certainty which species or groups of species were changing through time, we can say that arboreal vegetation in the sites we analyzed has not been stable over the last several thousand years in many of the studied sites (Figure 8). 

All the studied regions also show evidence of changes in palm abundances through time (Figure 8). All regions except Lake Zancudococha consistently showed large variations in the abundances of conical palm phytoliths (Figure 8). *Iriartea deltoidea* and *Socratea exhorriza* are two of the commonest palm species found in northwestern Amazonia, including at Yasuní, Tiputini, and around Lake Añangucocha [7,74], which produce these conical palm phytoliths. The conical palm phytoliths showed large magnitudes of change values (>10%), indicating large-scale fluctuations in the abundances of *I. deltoidea* or *S. exhorriza*. The trend of change values indicated that, at Yasuní and Tiputini, these taxa have increased in abundance through time and are more common in the modern landscape than they were in the past (Figure 8). The opposite pattern is evident at Lake Añangucocha, where these palms had negative trends of change values, indicating that they were less common in the modern landscape than they were in the past. The increasing abundance of these palm taxa at Yasuní and Tiputini may have been associated with past fire events (Figure 8). Though *I. deltoidea* and *S. exhorriza* are both fire-sensitive palms, it has been shown that the successional pathways following site abandonment can lead to significant increases in their abundance (especially *I. deltoidea*) [34]. Interestingly, this recovery of *I. deltoidea* did not seem to occur at Lake Añangucocha, which may be because the site was used more heavily and populations may have been more severely depleted. *I. deltoidea* is commonly used for construction, and sites with heavy human land use can show depleted populations that do not recover [27,32].

The northwestern Amazonian palms that produce spherical echinate phytoliths (Figure 8) include species of *Oenocarpus*, *Attalea*, *Phytelephus*, and *Mauritia* [47,48,75]. The abundances of these palms have also fluctuated significantly through time, though not to the degree of the palm taxa that produce conical phytoliths. The trends of change in these spherical echinate-producing genera, however, vary between and within regions. These genera have mostly increased through time at Yasuní (Figure 8). *Oenocarpus bataua* is the commonest palm species producing spherical echinate phytoliths in the modern landscape at Yasuní, and its abundances are higher now than in the past. *O. bataua* is a palm that is widely used for its fruit both in pre-Columbian and modern times [26], and its abundances may have been enriched as a result of past and present human activities. The spherical echinate-producing palm species at Tiputini, which is located <25 km from Yasuní, show the opposite trend (Figure 8). *O. bataua* is also the most common species at Tiputini that produces spherical echinate palm phytoliths, and its abundance is likely lower now than it was in the past. At Lake Añangucocha, the abundances of spherical echinate-producing palms have increased in some sites and decreased in others (Figure 8). Though there are no vegetation surveys for our soil core sites, forest plots surveyed within five kilometers of the lake show that *Attalea* (previous called *Scheelea*) and *Phytelephus* are the most common palm genera producing spherical echinate phytoliths. The core with the largest decrease of these spherical echinate palm phytoliths, also contained large amounts of charcoal (Figure 8), suggesting that fire sensitivity may have reduced the abundance of these palm genera at Lake Añangucocha. 

The areas around Lake Ayauch^i^, in the southeastern portions of the Ecuadorian Amazon, were also analyzed for charcoal and phytoliths [58]. The sediments collected from Lake Ayauch^i^ show that maize cultivation began in the region over 6000 years ago, and the area around the lake has been continuously occupied for thousands of years [33,49]. There was abundant charcoal in the soil cores, and ^14^C AMS dates showed that most of the documented fires occurred between 3000 and 1000 years ago [58]. The charcoal from the lake sediment core also reflected this pattern [33]. The phytoliths in soil cores collected around Lake Ayauch^i^ documented substantially more forest openings than we found at any of our five studied sites [58]. Unfortunately, the recent advances in the identification of palm phytoliths [47,48] were not available at the time of the phytolith analysis of soil cores collected around Lake Ayauch^i^, and we were not able to reconstruct changes in palms through time. However, the pollen record from the lake sediment contained very low amounts of palm taxa throughout the 3000 years of continuous occupation and fires [33]. This contrasts with palm pollen found in the sediments from nearby Lake Kumpak^a^, which showed that *Iriartea* palms increased significantly after site use and subsequent site abandonment [34]. 

Many of the hyperdominant species [8] include trees that produce rugose and ornate arboreal phytoliths and palms (where all produce phytoliths). Our results (Figure 3, Figure 4, Figure 5, Figure 6, Figure 7, Figure 8 and Figure 9) and previous work on soil cores and lake sediments analyzed in northwestern Amazonia [33,34,50,51,59,60] suggest that vegetation has not remained constant over the late Holocene and that modern hyperdominance captures only a snapshot in time. Instead, hyperdominance across this region likely changes through time and is a function of both climatic change and past human activity, including various forms of fire and forest management. It is likely that human influences on changes in hyperdominance have occurred in the more accessible areas, such as near major rivers or in the more seasonal forests, where past human activity was strongest [76].

## 4. Materials and Methods

### 4.1. Site Description and Data Collection

Our study includes four newly studied regions in the lowland Amazonian rainforests in Ecuador: the areas around Lakes Añangucocha and Zancudococha, and the forest inventory plots of Iamoe and Tiputini. We place these results in the context of our previous findings from the Yasuní forest plot (Figure 1). The sampling was not equivalent in all sites because the data were collected for different purposes. The charcoal and phytolith data from the soil cores collected in the forest plots will be paired with vegetation data collected in botanical surveys, whereas the soil cores collected around Lakes Añangucocha and Zancudococha will be paired with vegetation and geochemical reconstructions derived from lake sediment cores. We used all the data from the soil cores collected at these sites to reconstruct local scale variability in fire, disturbance, and vegetation change through time based on charcoal abundances and phytolith percentages.

Lake Añangucocha (−0.524306° S, −76.437629° W) is ca. 500 m × 500 m in size and lies 222 m above sea level (hereafter masl). The outflow of Lake Añangucocha runs for ca. 3.5 km into the Napo River, which feeds into the main Amazon River. Annual precipitation around the lake is approximately 2700 mm [77]. Fossil pollen and sedimentological analyses on a core raised from the center of the lake in 1982 provided a ca. 3600 year history of the site [71]. *Iriartea* pollen abundances rose ca. 2000 years ago, fell ca. 1000 years ago, and rose again 500 years ago—with elevated abundances persisting until the present [71]. The sediments were not analyzed for charcoal fragments, so the relationships between these changes in palm abundances and past human activity could not be assessed. Within 2 km of the lake, we collected 10 soil cores that fell into three general locations (Figure 1). Each 10 cm diameter soil core was collected in 10 cm depth increments with total core depths ranging from 40 to 80 cm. Surface samples of soil, which should contain phytoliths representative of modern vegetation, were also collected from each soil core location. There is, however, no vegetation survey to date that has been performed in the soil core locations.

Lake Zancudococha (−0.592044° S, −75.485009° W) is ca. 2 km in size, 185 masl, and lies ca. 3 km south of the Aguarico River (Figure 1). The outflow of the lake flows into the Aguarico River. Annual precipitation around the lake is approximately 2942 mm [77]. We collected 7 soil cores from two sides of the large lake. The soil cores on the northeast side of the lake range from flooded forests near the lake edge to terra firme forests that are 3 km from the lake (Figure 1). The soil cores on the southwest side of the lake were collected within 1 km of the lake and near the modern construction of a lodge (recently abandoned at the time of core collection in 2019). Each soil core was collected in 10 cm depth increments with total core depths ranging from 50 to 80 cm. Surface samples of soil were also collected from each soil core location. There are no vegetation data for the areas around the collected soil cores.

The Iamoe forest plot (−0.69° S, −76.73° W) consists of two 1 ha forest plots close to one another in the Yasuní National Park, Ecuador (Figure 1). The plot was established in 2021 by Juan Guevara-Andino and María José Endara, and all trees with a diameter at breast height (DBH) > 10 were tagged and measured. Voucher specimens were collected for every tree species recorded in the two plots, and species identification was performed by comparing these vouchers with botanical material deposited in Herbario Nacional del Ecuador (QCNE), Herbario Universidad Católica (QCA) and digital images from the Chicago Field Museum (https://plantidtools.fieldmuseum.org/es/rrc/5581). The plots are located in terra firme forests on clayed soils, with a highly dissected topography. The most abundant tree species in the two plots are *Chlorocardium venenosum* (Lauraceae), *Oenocarpus bataua* (Arecaceae), and *Iriartea deltoidea* (Arecaceae). Approximately 9% of the individuals measured in the plot were palms (Arecaceae). The annual precipitation is around 2826 mm (Valencia et al., 2004 [55]). One soil core with a corresponding surface sample from each plot was collected with a soil auger in 10 cm intervals until a depth of 80 cm was reached. 

The Tiputini forest plot (−0.63° S, −76.14° W) is located on the forest summits next to the Tiputini River on the edge of Yasuní National Park in Ecuador (Figure 1). The plot holds records for local amphibian, reptile, and bat diversity (Bass et al., 2010 [5]; Rex et al., 2008 [64]). receives 3252 mm of annual precipitation and has virtually no dry season (Malhi et al., 2004 [78]). The forest plot has a size of 1 ha in a square shape and contains old-growth, terra firme forests. Every individual tree with a DBH >10 was identified in censuses in 1997, 2002, 2007, 2010–2011, and 2019. Ten soil cores collected in January 2019 were distributed across the plot with a minimum distance of 10 m from the plot edge and each other. The soil cores were collected in 10 cm increments until a depth of 80 cm was reached. Surface samples for each core were collected by taking pinches of topsoil in a five-meter radius of the soil core. Approximately 11% of the surveyed trees are palms (Arecaceae), and the most common palm species is *Iriartea deltoidea*. 

The 50 ha Yasuní Forest Dynamics Plot (hereafter Yasuní) (−0.68° S, −76.40° W) was established in 1995 [55]. The plot is part of the ForestGEO botanical inventory network and is one of the most heavily studied in the world. Climatic conditions are similar to those listed for Tiputini. Individual trees >1 cm DBH (diameter at breast height) were measured in the western 25 ha of the plot, and all stems >10 cm DBH were measured in the eastern 25 ha of the plot [56]. Over 150,000 trees and 1104 species have been recorded in the plot [56]. Most of the plot, with the exception of the southwest corner, is comprised of mature forest species [55]. Ceramics have been recovered near the northwestern corner of the plot and are believed to be 500–1000 years old [55,79]. In 2018, we collected 25 soil cores (with corresponding surface samples) (Figure 1) in 10 cm depth intervals to a total depth of 80 cm. The fire history based on charcoal data was reported from 17 randomly selected soil cores, and the vegetation history based on phytolith data has been previously reported from 10 soil cores [50]. Approximately 2% of the species surveyed at the Yasuní forest plot are palms, and those individuals are primarily from the species *Iriartea deltoidea*.

### 4.2. Laboratory Processing of Charcoal and Phytoliths from Soil Cores

We isolated charcoal fragments from each depth interval of each soil core from all sites using standard laboratory techniques [57,80]. The organic materials within soils were deflocculated with detergent (e.g., Alconox) or a mild solution of hydrogen peroxide (<3% H_2_O_2_). After deflocculation, soils were sieved through a 500 μm mesh and charcoal was isolated from other soil particles. Charcoal was identified based on color, opacity, and angle [81], and the identified fragments were photographed using Image J ver. 1.54 [82]. For each sample (each depth interval of each core), the images were used to calculate the surface area (mm^2^/cm^3^) and volume (mm^3^/cm^3^) of charcoal standardized to the initial volume of soil [80].

Phytoliths were prepared from a 1 cm^3^ subsample from the soil samples using standard laboratory techniques that included deflocculation, clay removal via decanting, and floatation of the phytoliths using heavy density separation [39]. Phytoliths were mounted on microscope slides using Naphrax mounting medium (Naphrax, Chippenham, UK), and at least 200 arboreal or 300 total phytoliths [83,84] were counted for each sample. Phytolith counting was performed using Zeiss AxioScopes (Zeiss, Oberkochen, Germany) and 630× or 1000× magnifications. Extended scans for larger arboreal phytoliths or maize phytoliths [59,72,85] were performed at 200×. Phytolith identification was performed using published reference photographs [39,47,48,72,75] and the phytolith reference collection of the University of Amsterdam.

### 4.3. Data Analysis

All identified charcoal fragments over 1 mm^3^ were submitted to the DirectAMS Laboratory (Seattle, WA, USA) for radiocarbon (^14^C) dating to provide ages of past fire events. We used summed probability analysis to calculate the most likely timing of past fires and the time since the most recent fire for each of the five study regions using the ‘BchronDensity’ function in the *Bchron* package [86] for R ver. 4.3 [87]. We used the charcoal abundances to calculate the proportion of cores within a site that contained charcoal and the proportion of samples within a site that contained charcoal. The charcoal volumes (mm^3^/cm^3^) calculated in the depth intervals and the charcoal metrics for the soil cores and sites were used to compare with phytolith assemblages. 

Phytolith counts of the various morphotypes found within a depth interval were transformed to percentages of the total phytolith count to standardize and facilitate comparisons between depth intervals, cores, and regions. Because the phytolith taxonomic resolution of the four new sites was more detailed than achieved in the previous study of the Yasuní forest plot, we grouped some of the grass, palm, and arboreal morphotypes to standardize identification across all sites (Table S1). Detrended correspondence analysis, which is the most common ordination technique used on paleoecological datasets, was performed to assess turnover in phytolith assemblages between (i) depth intervals within a core, (ii) cores within a region, and (iii) cores across all regions. We also calculated the magnitude of change (maximum %–minimum %) and trend of change (basal %–surface %) [88] in selected phytolith types for all of the soil cores. These calculations allow quantitative comparisons of change in phytolith abundances, and by inference, the plant types associated with them through time within each soil core. The magnitude and direction (loss or gain) of phytolith change were compared with charcoal metrics to assess the degree to which fires drove past vegetation change. All data compiling, processing, and statistical analyses were performed using RStudio ver. 2024.04.2 [87].

## 5. Conclusions

The charcoal and phytolith data from the five regions of the Ecuadorian Amazon show the presence of low-level human activity that has occurred primarily over the last 2000 years. Evidence of past fire events occurred in all regions, and four of five contained evidence of modern fire. Maize cultivation was found at only one site (Añangucocha), a lake situated ca. 5 km from the Napo River (which leads into the Amazon River). Localized forest opening has occurred in the past in most regions. Palm abundances have changed over time, illustrating that the patterns of hyperdominance observed in today’s northwestern Amazonian forests are a snapshot in time.

## Figures and Tables

**Table 1:** ^14^C AMS radiocarbon ages based on individually dated charcoal fragments collected across the five study regions. Uncalibrated ages with standard error, and calibrated ages with 2-sigma ranges are shown in years before present (cal BP). Asterisks (*) in the uncalibrated date columns indicate where modern ages were obtained. The amount of modern carbon in the sample is shown, which was transformed to a modern calibrated age using the ‘pMC.age’ function of the *intcal* package for R [52]. Negative numbers for calibrated ages indicate the number of years since radiocarbon dating began (AD 1950).

Region	Core	Depth (cm)	Uncalibrated Date ± Std Error	Calibrated Ages ± 2 Sigma Range (cal BP)
Anangucocha	G	30–40	821 ± 20	714 ± 20
Anangucocha	G	40–50	915 ± 21	838 ± 48
Anangucocha	I	0	* 103.91 ± 0.24	−63 (AD 2013)
Zancudococha	F	20–30	1171 ± 22	1091 ± 50
Zancudococha	F	NA	1211 ± 25	1128 ± 45
Zancudococha	1	10–20	* 110.85 ± 0.25	−48 (AD 1998)
Iamoe	A	50	2572 ± 27	2701 ± 64
Iamoe	B	20	2556 ± 25	2670 ± 77
Tiputini	A	10–20	3709 ± 27	4046 ± 51
Tiputini	A	20–30	4233 ± 33	4771 ± 67
Tiputini	A	30–40	3695 ± 24	4033 ± 46
Tiputini	C	20–30	* 123.42 ± 0.30	−10 (AD 1960)
Tiputini	E	10–20	3405 ± 26	3646 ± 50
Tiputini	10	20–30	974 ± 23	860 ± 41
Yasuni	B	0–10	* 103.07 ± 0.29	−64 (AD 2014)
Yasuni	C	30–40	821 ± 25	717 ± 24
Yasuni	J	10–20	857 ± 21	754 ± 27

**Table 2:** Charcoal metrics for the sites used in the analysis. Proportion of cores containing charcoal, proportion of samples containing charcoal, and time since last fire (TSLF) per plot.

Region	Area (ha)	Num Cores (Charcoal)	Num Cores (Phytoliths)	Num Dated Fragments	TSLF (cal BP)	Prop Samples with Char	Prop Cores with Char
Añangucocha	>50	11	10	3	−63	0.22	0.36
Zancudococha	>50	10	7	3	−48	0.13	0.50
Iamoe	2	2	2	2	2670	0.72	1.00
Tiputini	1	10	8	6	−10	0.52	1.00
Yasuní	50	25	10	3	−64	0.26	0.76

## Data Availability

All data used in this study are provided in Supplementary Table S1.

## Supplementary Materials

The following supporting information can be downloaded at: https://www.mdpi.com/article/10.3390/plants13152048/s1, Table S1—Phytolith and charcoal data.
